# Exogenous pulmonary surfactant for acute respiratory distress syndrome in adults: A systematic review and meta-analysis

**DOI:** 10.3892/etm.2012.746

**Published:** 2012-10-15

**Authors:** LI-NA ZHANG, JUN-PING SUN, XIN-YING XUE, JIAN-XIN WANG

**Affiliations:** Respiratory Diseases Department, Chinese PLA General Hospital, Beijing 100853, P.R. China

**Keywords:** acute respiratory distress syndrome, pulmonary surfactant, mortality, meta-analysis

## Abstract

Acute respiratory distress syndrome (ARDS) is often characterized by reduced lung compliance, which suggests dysfunction of the endogenous surfactant system. The effectiveness of exogenous surfactants as replacements for the endogenous system in the treatment of ARDS in adults was assessed. Randomized controlled trials from Medline (1950–2011), Embase (1989–2011), the Cochrane Database of Systematic Reviews and the Cochrane Central Register of Controlled Trials (1994–2011) were analyzed. Two reviewers identified trials for inclusion and the results of included trials were quantitatively pooled with a fixed-effects model. Seven trials (2,144 patients) with good methodological quality were included in the analysis. Pulmonary surfactant treatment was not associated with reduced mortality [relative risk (RR), 1.00; 95% confidence interval (CI) 0.89–1.12]. Subgroup analysis revealed no reduced mortality for various surfactant types. Heterogeneity was not significant in the primary outcome analysis (I^2^=0%). There was no evidence of publication bias. Oxygenation, ventilation-free days, duration of ventilation and APACHE II scores did not undergo pooled analysis due to insufficient data. Exogenous surfactant did not reduce mortality in adults with ARDS in our meta-analysis, and we cannot accurately define whether exogenous surfactant has an effect on oxygenation from the included studies.

## Introduction

Acute lung injury (ALI) and acute respiratory distress syndrome (ARDS) are common, costly and potentially lethal diseases with mortality rates of ∼40%. They represent a spectrum of acute respiratory failure with diffuse, bilateral lung injury and severe hypoxemia caused by non-cardiogenic pulmonary edema. There are several clinical disorders associated with the development of ARDS, including sepsis, pneumonia, aspiration of gastric contents and major trauma (1). These pulmonary or extra-pulmonary insults may increase alveolar epithelial-endothelial permeability, lead to alveoli flooding, reduce lung compliance and deprive the lungs of adequate quantities of surfactant. However, no specific pharmacological therapy has proven effective for ARDS and therapy is mainly supportive with the use of lung-protective mechanical ventilation, which may limitedly improve survival but with high mortality (2). The effectiveness of pharmacological treatments (including corticosteroids, acetylcysteine, alprostadil, sivelestat sodium and pentoxifylline) is also not satisfactory and remains controversial (3,4).

Pulmonary surfactant is a lipoprotein complex consisting of phospholipids (90%) and surfactant-specific proteins (10%) produced by type II alveolar cells. Surfactant reduces alveolar surface tension, prevents alveolar collapse and enables gas exchange and alveolar ventilation at low transpulmonary pressures (5). One of the characteristics of ARDS is reduced lung compliance, implicating dysfunction or deficiency of the endogenous surfactant system. Bronchoalveolar lavage fluid from patients with ARDS has low concentrations of phosphatidylcholine, phosphatidylglycerol and surfactant-specific proteins (6). Therefore, treatment with an exogenous surfactant that may aid the restoration or replenishment of the depleted endogenous surfactant pool may improve ARDS outcome.

Intratracheal administration of exogenous surfactant is an effective therapy for premature neonates and children with acute respiratory failure (7). Exogenous surfactant may improve oxygenation, but it has not been shown to reduce mortality in adults with ALI/ARDS (8). One previous randomized multicenter trial failed to demonstrate any improvement in mortality and oxygenation following the bolus administration of exogenous natural porcine surfactant to patients with ALI/ARDS (9). In addition, another trial showed that recombinant surfactant protein C (rSP-C)-based surfactant was of no clinical benefit to patients with severe direct lung injury (10). However, a multicenter study showed that early administration of Surfactant-BL (bovine lung extract surfactant) led to reduced mortality in cardiac patients who developed ARDS postoperatively (11).

Since the effectiveness of exogenous surfactant administration in adults with ARDS remains unclear, in the present study we performed a meta-analysis to analyze the effects of exogenous surfactant treatment on 28–30-day mortality to address this issue.

## Materials and methods

### Trial identification and search strategy

We used systematic methods to identify randomized controlled trials that compared administration of exogenous pulmonary surfactant with an appropriate control group (standard therapy or placebo) for adults diagnosed with ARDS. Trials that reported mortality and/or pulmonary physiological parameters and that used objective diagnostic criteria of ARDS were included. We excluded studies reporting only physiological endpoints, studies in children, abstracts, case reports, editorials, nonhuman studies and reports not in English.

To identify all the relevant trials, we searched Medline (1950-July 2011), Embase (1989-July 2011), the Cochrane Database of Systematic Reviews and the Cochrane Central Register of Controlled Trials (1994–2011), with a search strategy combining medical subject headings and key words: <‘adult respiratory distress syndrome’, ‘acute respiratory distress syndrome’, or ‘ARDS’>; <‘pulmonary surfactant’ or ‘lung surfactant’>; and <‘adult’>.

### Trial selection and quality assessment

**Figure 1:**
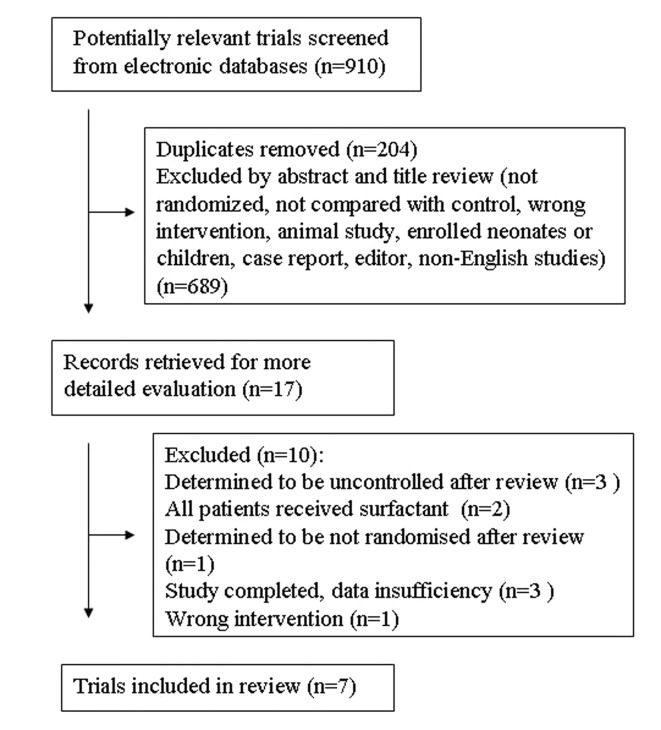
Flowchart of identification of studies included in the review.

Two reviewers (L.N.Z. and J.P.S.) independently assessed the eligibility of each study and resolved disagreements by consensus. Candidate studies for inclusion were obtained and reviewed in detail as indicated (Fig. 1).

Quality assessment of these studies was performed by two investigators using a 10-point scoring system modified from a previous meta-analysis (12). For each trial, we evaluated the following aspects: methods of randomization, allocation concealment, blinding, inclusion and exclusion criteria defined, similar baseline at study entry, treatment protocol clearly described, co-intervention that may affect outcome, outcome definition, extent of follow-up described clearly and intention-to-treat (ITT) analysis.

### Data abstract and outcome measures

Two reviewers (L.N.Z. and J.P.S.) independently abstracted data of relevant outcome measures with a standardized spreadsheet. Disagreements were resolved by consensus among authors.

The primary outcome measure was mortality 28–30 days after randomization. Secondary outcome measures included the oxygenation index (PaO_2_:FiO_2_ ratio), the number of ventilation-free days, and the mean duration of ventilation. Finally, we assessed the adverse events, including hypoxia and hypotension.

### Statistical analysis

We conducted a meta-analysis with a fixed-effects model using Review Manager (RevMan) 5.0 software, unless there was significant heterogeneity, and considered P≤0.05 (two-sided) to indicate a statistically significant result. We reported binary outcomes as risk ratios (RRs) and continuous outcomes as weighted mean differences. A Z-test was performed to statistically evaluate the treatment effects in different groups (13). Moreover, we assessed heterogeneity between studies for each outcome using the I^2^ measure, and considered an I^2^ value >50% to indicate substantial heterogeneity (14). A statistical test for funnel plot asymmetry was used to investigate the publication bias.

## Results

### Trial flow

By searching electronic bibliographic databases, we identified 910 citations, of which 204 were duplicate reports. Next, we excluded 689 citations with irrelevant titles or abstracts to obtain 17 studies, of which 7 met the criteria for review after detailed evaluation (9,10,15–19) (Fig. 1). Reviewers agreed on all studies for inclusion.

### Study characteristics

The seven included studies published between 1994 and 2011 were multicenter, randomized and controlled trials, in which 2,144 patients were enrolled. Different types and doses of surfactant were used in these trials. Two trials used synthetic surfactant at different doses but without recombinant surfactant proteins (15,16). One trial used modified natural bovine surfactant containing surfactant proteins B and C (17). One trial employed modified natural porcine surfactant containing surfactant proteins B and C (9). Three trials used synthetic surfactant containing rSP-C at the same dose (10,18,19). A variety of interventions were used in the control groups, including standard therapy without placebo, or a placebo of 0.6 or 0.45% saline. Two studies included patients only with sepsis-related ARDS (15,16). The other studies included patients with direct lung injury (aspiration of gastric contents and pneumonia) and indirect lung injury (trauma or surgery, multiple blood transfusions, burn injury, pancreatitis and toxic injury). A total of 418 patients with ALI/ARDS were included in the study by Kesecioglu *et al*(9), but only 327 patients (78.2%) had ARDS at baseline. Due to similar issues in the study by Spragg *et al*(10), 440 patients without ARDS at baseline were excluded from these studies. Baseline characteristics of the patients are presented in Table I. All trials had high methodological quality and low risk of bias (Table II).

### Primary outcome: mortality (28 to 30 days)

**Figure 2:**
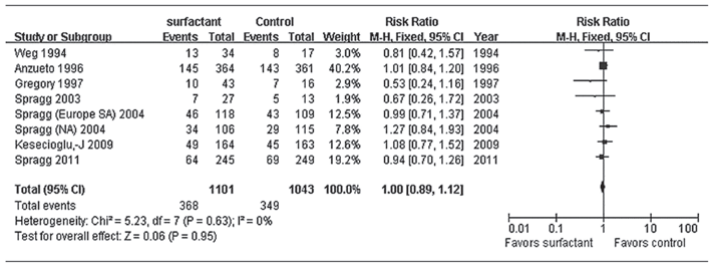
Forest plot depicting risk ratio (RR) with 95% confidence interval (95% CI) for 28–30-day mortality in patients treated with surfactant compared with controls. The summary mortality estimate is RR 1.00 (95% CI, 0.89–1.12). I^2^=0%, indicating no substantial heterogeneity. M-H, Mantel-Haenszel.

**Figure 3:**
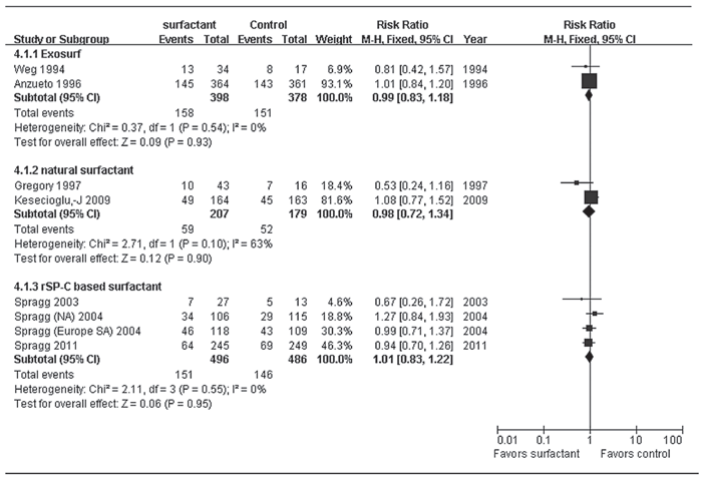
Subgroup analyses for 28–30-day mortality based on various types of surfactant: synthetic surfactant without surfactant protein (Exosurf), modified natural surfactant, and rSP-C-based surfactant. rSP-C, recombinant surfactant protein C; 95% CI, 95% confidence interval; M-H, Mantel-Haenszel.

**Figure 4:**
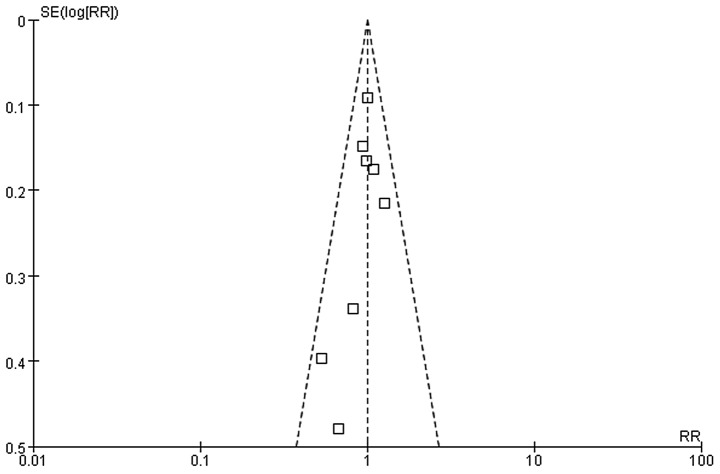
Funnel plot inspection of 28–30-day mortality. RR, risk ratio.

According to all the seven trials, the difference in 28–30-day mortality between the surfactant and control groups was not statistically significant (P=0.95). Treatment with pulmonary surfactant was not associated with reduced mortality compared with controls [RR, 1.0; 95% confidence interval (CI), 0.89–1.12; Fig. 2]. As there was no evidence of heterogeneity (I^2^=0%), we assessed the data using a fixed-effects model. In the subgroup analysis, we found no difference among various types of surfactant: synthetic surfactant without surfactant protein (exosurf; RR, 0.99; 95% CI, 0.83–1.18), modified natural surfactant (RR, 0.98; 95% CI, 0.72–1.34) and rSP-C based surfactant (RR, 1.01; 95% CI, 0.83–1.22) were statistically indistinguishable (Fig. 3). For our primary outcome, we found no significant funnel plot asymmetry based on visual inspection, suggesting no evidence of publication bias (Fig. 4).

### Secondary outcomes

The included trials all showed changes in PaO_2_/FiO_2_ ratio following treatment with surfactant, although the data forms and monitoring time points were different. Some data was incomplete and thus difficult to analyze with combined statistics. Only half of the patients in one trial showed significant improvements in PaO_2_/FiO_2_ ratio at 24 h after surfactant treatment (19), while the other trials showed no improvement in PaO_2_/FiO_2_ ratio. The outcomes of ventilation-free days, mean duration of ventilation and APACHE II scores could not be pooled and analyzed due to insufficient data. Moreover, no trial showed improvement of these indicators in the surfactant group.

### Adverse events

Most trials reported that surfactant therapy was well tolerated, and no patient was withdrawn from any trial due to adverse events. Hypotension and hypoxia were the most commonly reported adverse events in the included studies. Six trials reported hypoxemia (9,10,16–19) and four reported hypotension (9,16,17,19). The following adverse events were reported in only one trial each: increased secretions (16), acidemia, air leak, bronchospasm, decreased consciousness, oxygen desaturation, premature ventricular contractions, body rash, renal failure, shock (17), supraventricular tachycardia (18), bradycardia (19) and airway obstruction (10).

## Discussion

Although a multitude of causes may lead to ARDS, the reduction in the amount and function of endogenous surfactant is a shared characteristic (20). Exogenous surfactant replacement therapy may help restore or replenish insufficient endogenous surfactant activity, thereby improving the ARDS outcome. Our meta-analysis suggested that administration of exogenous surfactant did not reduce 28–30-day mortality in adults with ARDS (RR 1.0; 95% CI, 0.89–1.12). Furthermore, subgroup analysis showed that all preparations of surfactant similarly failed to reduce mortality. There were insufficient data available for analysis of changes in oxygenation, APACHE II scores and ventilation characteristics.

**Figure 5:**
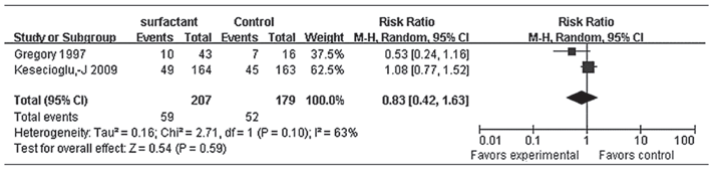
Subgroup analyses of 28–30-day mortality after treatment with modified natural surfactant (random-effects model). 95% CI, 95% confidence interval; M-H, Mantel-Haenszel.

Heterogeneity of the primary outcome of 28–30-day mortality in our study was low (I^2^=0%). The surfactants used in the included trials consisted of synthetic surfactant without surfactant protein (Exosurf), modified natural surfactants and rSP-C-based surfactant. These surfactants may have had different effects on outcomes due to differences in the composition of phospholipids and surfactant proteins, so we performed subgroup analysis. Exosurf and rSP-C-based surfactant subgroups exhibited low heterogeneity (I^2^=0%), while the modified natural surfactant subgroup had higher heterogeneity (I^2^=63%). Gregory *et al* demonstrated that bovine lung extract surfactant at 100 mg/kg LBW (maximum 4 or 8 doses) improved survival compared with the control group (17), whereas Kesecioglu *et al* found no benefit in outcome with the administration of exogenous natural porcine surfactant (9). The differences between these two trials may be caused by the use of different natural surfactant types. Thus, we applied a random-effects model (Fig. 5) and these results did not show significantly reduced mortality (RR, 0.83; 95% CI, 0.42–1.63) either.

Our methods minimized bias by including a comprehensive search strategy, abstracting data in duplicate, using a predefined protocol outlining our hypotheses and including methodological assessment of primary studies and planned statistical analyses. All included trials had a high methodological quality with scores between 8 and 10. Funnel plots showed low risk of bias.

Davidson *et al* reported that exogenous surfactant may improve oxygenation, but they detected no improvement in mortality (8). Oxygenation outcomes were only included in two trials, both of which used rSP-C-based surfactant, and did not achieve statistical significance. We included two additional trials to compare with their meta-analysis, and generated additional subgroup analyses to estimate the treatment effects more precisely. Our results were consistent with theirs and indicated that exogenous pulmonary surfactant has no significant effect on 28–30-day mortality. Although the oxygenation outcome could not undergo pooled analysis due to insufficient data, no improvement in oxygenation was observed in the newly included trials.

We found that the natural surfactant subgroup had higher heterogeneity. The trial that used bovine lung extract surfactant showed significantly decreased risk of 28–30-day mortality (17). One meta-analysis demonstrated that bovine lung extract surfactant may significantly decrease mortality in children with acute respiratory failure (7). A multicenter study that used a historical control group for comparison showed that early administration of Surfactant-BL (bovine lung extract surfactant) leads to reduced mortality and marked improvement in oxygenation at 24 h in cardiac patients who develop postoperative ARDS (11). However, trials in adults indicated that surfactant is not effective in decreasing mortality. This is probably due to differences in the etiologies of lung injury in adults and children, design features of different trials, the mode and timing of surfactant administration or the type and dose of surfactant used (7). Therefore, further randomized controlled studies of bovine lung extract surfactant in treatment for adults with ARDS are required.

Our meta-analysis suggests that exogenous surfactant does not reduce 28–30-day mortality in the treatment of adults with ARDS. Nevertheless, we are unable to accurately define the effects of exogenous surfactant on oxygenation from the included studies. Therefore, clinicians who employ exogenous surfactant to treat adult patients with ARDS should use it cautiously.

## Figures and Tables

**Table I:** Baseline characteristics of the eligible trials.

First author, year (ref.)	No. of patients	Age (years)	Gender (male %)	Type of surfactant	Surfactant dosing	Delivery method	Treatment duration	Initial PaO_2_/FiO_2_	Initial APACHE II score	Predisposing event
Weg 1994 (15)	51	C, 51±19	C, 29	Exosurf (synthetic no surfactant protein)	G 1, 21.9 mg DPPC/kg/day	Aerosolized	Max.	C, 146.5±20.4	C, 14.2±6.4	Sepsis
	C, 17	G 1, 51±20	G 1, 41		G 2, 43.5 mg DPPC/kg/day		120 h	G 1, 124.2±11.8	G 1, 16.5±6.7	
	S, 34	G 2, 51±17	G 2, 82					G 2, 161.5±16.2	G 2, 15.7±6.6	
Anzueto 1996 (16)	725	C, 53±18	C, 58	Exosurf (synthetic no surfactant protein)	112 mg DPPC/kg/day	Aerosolized	Max.	C, 140±64	Not available	Sepsis
	C, 361	S, 50±17	S, 59				5 days	S, 145±82		
	S, 364									
Gregory 1997 (17)	59	C, 40±18.1	C, 62.5	Bovine lung extract (containing SP-B.C)	G 1, 50 mg/kg^a^	Intratracheal	Max.	C, 128 (71–286)	Not available	Trauma, aspiration, transfusions, sepsis
	C, 16	G 1, 39.1±13.2	G 1, 50		G 2, 100 mg/kg^b^		96 h	G 1, 98 (84–402)		
	S, 43	G 2, 42.7±11.4	G 2, 75		G 3, 100 mg/kg^a^			G 2, 124 (40–234)		
		G 3, 42.8±15.4	G 3, 68.4					G3, 133 (77–401)		
Spragg 2003 (18)	40	C, 51±5	C, 38.4	Venticute (rSP-C-based surfactant)	G 1, 1 ml/kg^b^	Intratracheal	24 h	C, 120.9±6.5	C, 10.9±1.1	Burn, aspiration, sepsis, pneumonia, trauma, pancreatitis
	C, 13	G 1, 59±5	G 1, 53.3		G 2, 0.5 ml/kg^b^			G 1, 133.6±8.9	G 1, 10.2±1.2	
	S, 27	G 2, 52±5	G 2, 33.3					G 2, 113.9±8.3	G 2, 10.1±1.7	
Spragg 2004 (19)^d^	221	C, 53.1±17.6	C, 64	rSP-C-based surfactant	1 ml/kg^b^	Intratracheal	24 h	C, 130±39	C, 17.9±6.6	Trauma, aspiration, transfusions, sepsis, burn, toxic injury
	C, 115	S, 56.5±17.8	S, 61					S, 132±40	S, 18.6±6.1	
	S, 106									
Spragg 2004 (19)^e^	227	C, 53.0±18.0	C, 72	rSP-C-based surfactant	1 ml/kg^b^	Intratracheal	24 h	C, 136±39	C, 16.6±5.8	Trauma, aspiration, transfusions, sepsis, burn, toxic injury
	C, 109	S, 50.6±17.5	S, 68					S, 137±40	S, 17.4±7.5	
	S, 118									
Kesecioglu 2009 (9)	327	C, 57.4±15.7	C, 65.7	Natural (porcine) (containing SP-B.C)	600 mg/kg^c^	Intratracheal	36 h	C, 161.4±55.2	C, 25.2±7.3	Sepsis, trauma, aspiration, shock, pneumonia
	C, 163	S, 57.2±15.9	S, 63					S, 156.7±54.8	S, 25.7±8.2	
	S, 164									
Spragg 2011 (10)	494	C, 56.5±0.83	C, 67.2	rSP-C based surfactant	1 ml/kg^a^	Intratracheal	96 h	C, 124.1±1.32	C, 17.8±0.32	Aspiration, pneumonia
	C, 249	S, 57.5±0.8	S, 66.1					S, 123.8±1.3	S, 18±0.33	
	S, 245									

C, control; S, surfactant; G, group; rSP-C, recombinant surfactant protein C; SP-B.C, surfactant protein B and surfactant protein C. Maximum of

a 8,

b 4,

c 3 doses.

d North American;

e European and South African.

**Table II:** Methodological quality scores.

First author, year (ref.)	Randomization	Allocation concealment	Blinding	Inclusion and exclusion criteria defined	Similar baseline at study entry	Treatment protocol clearly described	Cointervention that may affect outcome	Outcome definition	Extent of follow-up described clearly	ITT analysis	Final score
Weg 1994 (15)	1	1	1	1	1	1	1	1	1	1	10
Anzueto 1996 (16)	1	1	1	1	1	1	1	1	1	1	10
Gregory 1997 (17)	1	0	0	1	1	1	1	1	1	1	8
Spragg 2003 (18)	1	1	0	0	1	1	1	1	1	1	8
Spragg 2004 (19)	1	0	1	1	1	1	1	1	1	1	9
Kesecioglu 2009 (9)	1	1	0	1	1	1	1	1	1	1	9
Spragg 2011 (10)	1	1	1	1	1	1	1	1	1	1	10

Score 0 if not described or if description is inadequate or unclear and 1 if appropriately described. ITT, intention to treat.
